# The Influence of Tourists’ Experience of Quality of Street Foods on Destination’s Image, Life Satisfaction, and Word of Mouth: The Moderating Impact of Food Neophobia

**DOI:** 10.3390/ijerph17010163

**Published:** 2019-12-25

**Authors:** Sangmook Lee, Hyebin Park, Yoonyoung Ahn

**Affiliations:** 1 Department of Hospitality and Tourism Management, Kyungsung University, 309 Suyeong-ro, Nam-gu, Busan 48434, Korea; mookvndn@ks.ac.kr; 2 Fisheries Outlook Center, Korea Maritime Institute, 26, 367 Haeyang-ro, Yeongdo-gu, Busan 49111, Korea; heabin_p@naver.com; 3 Department of Hotel and Tourism Management, Sejong University, 209 Neungdong-ro, Gwangjin-gu, Seoul 05006, Korea

**Keywords:** experience quality, destination image, life satisfaction, WOM (word of mouth), food neophobia

## Abstract

Due to growing food-related tourism, there is increasing interest about street foods worldwide, including South Korea. Many types of food-related experiences have been considered as one of the significant elements to develop positive perceptions about a destination, and street food has been recognized as a critical clue for encouraging tourists to a destination. Previous scholars mentioned street food as a public health risk element as well as a significant factor to attract tourists’ attention. Therefore, this study aims to find out how experiential quality of street foods is related to the destination image, life satisfaction, and word of mouth as perceived by tourists in night markets of South Korea. Data was collected from 325 foreigners who visited night markets and have experienced street foods in Korea. This study demonstrates the results of the influence of quality of street foods on tourist experience, on destination image, on life satisfaction, and on word of mouth in Korea. In addition, the result shows a moderating impact of food neophobia on the formulated relationships. There are statistically significant differences between groups with high neophobia perception and low neophobia perception of street foods. Based on the results of this study, we propose not only academic implications for future studies, but also managerial implications for food enterprises and food tourism organizers related to street food.

## 1. Introduction

Food has been considered as an important attraction for travelers, especially those who are traveling abroad. As food tourism grows, food is becoming an important part of the marketing strategy of places of destination [1]. Therefore, many destinations are trying to introduce or develop exotic foods to attract tourists by providing various culinary experiences [2]. According to Boyne, Williams, and Hall (2002) [3], travelers typically spend approximately 40% of their costs in enjoying food in the destination. Because of growing food tourism, there is increased interest in street foods worldwide, including South Korea. The growth and expansion of street food may be attributed to the critical economic benefits derived from tourists, since street food has various strengths such as low cost, convenience, high nutrition content. It also gives positive provisions for employment and income [4,5,6,7]. Street foods are also a nearly universal phenomenon of urban life in many countries not only for developing counties or advanced economies in Asia including Japan and South Korea [8,9].

Perception or value is built by various experiences. Particularly, people reflect their experiences through various factors such as service excellence, playfulness, or customer return on investment [10]. These experiences can impact people’s perception of brand as well as satisfaction and loyalty directly [11,12]. In the tourism industry, hence, various experiences have been considered as one of the significant elements to develop positive perceptions about the destination, and street food has been recognized as a critical clue for encouraging tourists to visit a destination [6,7]. In view of the increased significance of experiential value in determining important outcomes, the managers or organizers in the street food industry need to know the critical elements that are closely related to tourists’ experiential clues and other significant factors. Namely, satisfied experience quality between a provider and its consumers will contribute to build a positive image as well as behavioral intentions [13]. Although we know the importance of improving tourists’ experiences, it is not simple to find an accurate way of measuring these because visiting a destination and enjoying the locale or street foods are not unique to current travelers anymore. People want more than just consumption of foods and services during their trip; therefore, they keep looking for special and memorable things through exotic experiences such as local culture with street foods [12,14]. Hence, tourism associations and governments must pay attention to the circumstances for improving tourists’ experiential qualities and creating memorable experiences during their trips by considering diverse factors such as food quality, service employee, and environmental elements.

Previously, scholars have conducted a number of studies to better understand the role of food consumption for tourists including food experiences in various festivals [15,16,17], food travel for experiencing local island foods [18,19], and urban tours for food [20,21,22]. Particularly, previous research on street foods have been mainly focused on the food safety for public health issues such as hygiene or disease from street foods [23,24,25] and the traveler’s knowledge of street foods [26]. Although research on food perception was conducted in previous studies, studies that consider various factors that may affect the street food experience for tourists are still insufficient. Especially, emotional factors, as well as physical factors, are important factors for travelers to choose street food, but research on these areas is also lacking. Therefore, this study considers the functional aspect (e.g., outcome quality and physical quality) as well as emotional aspect such as interpersonal quality from employees. More specifically, the current study attempts to verify the relationships among various perceived qualities from tourists and outcome variables (e.g., image of destination, satisfaction, and word of mouth). In addition, this study considers tourists’ personal characteristics in relation to food, neophobia, as an antecedent that can influence the formulated relationships to perceived quality from their street food experience, destination image, satisfaction, and word of mouth.

Therefore, this study aims to clarify the effect of experiential quality of street foods on destination image, life satisfaction, and word of mouth as perceived by tourists who visited a local market and experienced street foods. Information gathered from this study could be used by the state tourism officers in the development of strategic plans towards regulating street food handling to satisfy the visitors by considering various elements of street foods including emotional and functional nature.

## 2. Literature Review and Research Hypotheses

### 2.1. Influence of Experiential Quality on Destination Image

Basically, the basis of concept of experiential quality has been brought from service quality and affects tourists’ specific experiences [13]. Gronroos (1984) [27] defined service quality as the result of comparisons and evaluations between the services the customer expected at the service site and the services actually perceived. It also claims to be in a functional relationship with variables such as customer expectations, images, technical and functional quality. The ‘Nordic model’ of Gronroos (1984) [27] consists of the factors of service quality evaluation, service type and method, and corporate image. The ‘Third-order factor model’ of Brady and Cronin (2001) [28] was developed by integrating the existing ‘Three-component model’ [29] and the ‘Multi-level model’ [30]. It consists of three dimensions: quality of results, physical environment, and interaction. Specifically, the quality of the results evaluated the type of service and the waiting time, the physical environment quality used variables of the internal facilities, the design, the conditions of the surrounding environment, and the interaction quality variables consist of items such as the employee’s behavior, attitude and professionalism.

On-site experience can be mainly evaluated and represented as people’s perceived experiential quality based upon the comparison between expectation and actual performance. Previous studies employed different variables in order to measure experience quality in various service sector [31,32]. Otto and Ritchie) [32] developed elements to measure experience quality that comprised of four factors—peace of mind, hedonics, recognition, and involvement—from three different tourism service sectors: airlines, hotels, and tourist attractions. They reported that experience quality focused more on subjective concepts such as internal aspects. For Kao et al. (2008) [33], the quality of experience consisted of four main sub-dimensions: surprise, participation immersion, and fun-immersion. Pine et al. (1999) [34] and Ostrowski et al. (1993) [35] studied the customer experience and perception and they verified that the good experience over time following impressive experiences will lead to a positive image in their airline service research. Individuals’ perceptions about restaurant image is likely to fully reflect experiences of customers’ cumulative consumption such as food, service, and atmospherics in the restaurant industry. However, there is still little research related to the experiential quality of specific tourism participation [31,36]. Therefore, this study extended the experiential quality construct from the concept of service quality in the service sector. Several studies indicate three dimensions of quality which consist of outcome quality, interaction quality, and physical environment quality. Ryu and Han (2010) [37] examined the relationships among the three kinds of service quality dimensions, satisfaction, and behavioral intention in quick-casual restaurants. Chow et al. (2007) [38] also captured three dimensions of service quality (i.e., interaction quality, physical quality, outcome quality) and conducted them in restaurant research. De Rojas and Camarero (2008) [39] also demonstrated that experiential quality consisted of interaction quality, outcome quality, and physical environment quality. Therefore, this study considers the three dimensions of quality as experiential quality in our research model. In detail, the outcome quality consists of food related factors, the interaction quality encompasses service factors, and the physical environment quality is regarded as the environment factor. These factors are conceived of service quality factors. 

In this study, we investigated whether three kinds of experiential quality have a positive and direct effect on the destination image of tourists who have experienced Korean street food. There is some literature related to that relationship between the experiential quality of food and destination image. Mak et al. (2012) [40] examined tourists’ food experiences in the destination and the factors which can evaluate the dining experience in how the destination affects their satisfaction. Moreover, they stated that the research model of tourists’ food consumption behaviors and attitudes consist of tourists’ characteristics, the food in the destination and the destination environment. Kim et al. (2012) [41] demonstrated that the experience of enjoying Korean food leads to a positive national image which can be a driver for revisit intention to Korea for food tourism. Based on the aforementioned discussion, it is logical to posit the following hypotheses:

**Hypothesis** **1** **(H1).** *The outcome quality (the quality of food) has a positive and direct effect on the destination image.*

**Hypothesis** **2** **(H2).** *The interaction quality (the quality of service) has a positive and direct effect on the destination image.*

**Hypothesis** **3** **(H3).** *The physical environmental quality (the quality of environment) has a positive and direct effect on the destination image.*

### 2.2. Influence of Experiential Quality on Life Satisfaction

People compare their experience about a place/product with previous expectations, which can be a driver of affirmative or negative disconfirmation [33]. Affirmative or negative disconfirmation are the basis of tourist satisfaction or dissatisfaction [42]. In an experiential perspective, life satisfaction is defined as the result of tourists’ overall evaluation of the contents presented by not only service providers but also consumers. 

Experiential quality has been found as a predictor that is positively influenced on experiential value and satisfaction [31,33,43]. Wu and Li (2017) [36] and Jin et al. (2013, 2015) [43,44] also indicate that visitor’s experiential quality is an important predictor for creating image, experiential value, and satisfaction in the context of theme parks. To be more specific, Nield et al. (2000) [45] explored international tourists’ satisfaction regarding dining experiences and found factors that can affect overall satisfaction. They revealed that food quality has a significant influence on tourists’ overall satisfaction. Renko et al. (2014) [46] reported that the image of local food, which included quality and affective image of food, food uniqueness had positive effects on tourist satisfaction. Correia et al. (2008) [47] investigated that the tourists’ satisfaction of experience was statistically affected by the elements of food and atmosphere. Chi et al. (2013) [48] stated that food diversity and quality, and presentation are crucial factors on making positive food image both satisfaction and culinary quality perception. Sanchez-Cañizares and Castillo-Canalejo (2015) [49] found that several factors can lead to tourists’ motivations of gastronomy experiences and revisiting a place. The authors revealed that the perception of establishments’ atmosphere, service, and food quality were statistically different.

In summary, higher experiential quality perceived by experience in the destination could lead to both higher satisfaction levels and more affirmative behavioral intentions in common [50,51]. In this study, we investigated three kinds of experiential quality that have a positive and direct effect on the life satisfaction of tourists who have experience of Korean street food. On this basis, we propose the following hypotheses:

**Hypothesis** **4** **(H4).** *The outcome quality (the quality of food) has a positive and direct effect on life satisfaction.*

**Hypothesis** **5** **(H5).** *The interaction quality (the quality of service) has a positive and direct effect on life satisfaction.*

**Hypothesis** **6** **(H6).** *The physical environmental quality (the quality of environment) has a positive and direct effect on life satisfaction.*

### 2.3. Influence of Destination Image on Life Satisfaction and Word of Mouth

According to the Crompton (1979) [52] and Fakeye and Crompton (1991) [53]’s studies, destination image is defined as an individual’s cognitive representation of beliefs, knowledge, feelings, and overall perception of a particular destination. Destination image has two kinds of important roles in individuals’ behaviors. These are to effect the decision-making process of destination choice and after-decision-making behaviors including evaluation (satisfaction), participation (on-site experience), and behavioral intentions in future (revisit intention and word-of-mouth communication) [42,51,54,55]. This study examined the second role of the destination image, how destination image affects decision-making behaviors such as satisfaction and word of mouth. There are many previous studies showing the relationships between the destination image, satisfaction, and behavioral intentions. 

It has been commonly mentioned in the literature that image of destination has influence on tourists’ behaviors [42,51,53]. The tourist behaviors consist of a destination choice to visit and subsequent evaluations. The subsequent evaluations include overall satisfaction which is perceived during travel or experience in the destination, and the future behavioral intentions including the intention to revisit and word of mouth. Castro et al. (2007) [56] investigated that a destination image affects tourists’ future behavior through satisfaction. People having a favorable destination image would perceive their on-site experiences positively, which would lead to greater satisfaction levels and behavioral intentions [51]. In various sectors of hospitality and tourism, there is much research showing the relationships among image, satisfaction, and loyalty such as word of mouth and revisit intention. In the restaurant industry, previous studies demonstrated that the image of brand or place has significant influence on consumers’ satisfaction and revisit intention [57,58,59]. Andreassen and Lindestad (1998) [58] discussed organizations’ image influence on consumer behavior, especially loyalty of customers in the service dimension. Ryu et al. (2008) [59] revealed the effects of the quick-casual restaurant image on customer satisfaction, perceived value, and behavioral intentions. Thus, the following hypotheses are formulated:

**Hypothesis** **7** **(H7).** *The destination image has a positive and direct effect on the life satisfaction.*

**Hypothesis** **8** **(H8).** *The destination image has a positive and direct effect on word of mouth.*

### 2.4. Relationship between Life Satisfaction and Word of Mouth

Numerous studies found direct and affirmative relationships between customer satisfaction and behavioral intentions, such as repurchase, revisit, and word of mouth communication [37,60,61,62]. As suggested by Namkung and Jang (2007) [61], customer satisfaction and behavioral intentions are linked positively in mid-to-upscale restaurants. Kim et al. (2009) [62] also argued that satisfaction of the consumer has positive effects on the return intention and recommendation in university foodservice dining operations. Moreover, in previous studies related to tourism, satisfaction about place or destination has been discussed as crucial objects of behavioral intentions such as revisit intention or willing to recommend to other people [63,64,65,66,67,68]. Therefore, this study extended the life satisfaction construct from the concept of service satisfaction. We examined the relationship between satisfaction and word of mouth. According to this, we formulated the following hypothesis:

**Hypothesis** **9** **(H9).** *The life satisfaction has a positive and direct effect on word of mouth.*

### 2.5. The Moderating Role of the Level of Food Neophobia in the Relationship among Experiential Quality, Destination Image, Life Satisfaction, and Word of Mouth

Throughout history, humans with omnivorous characteristics have viewed eating novel and unfamiliar food as potentially harmful. Therefore, they have fears and reluctance to eat novel food [69]. Food neophobia has been widely used to predict the willingness to try to eat unfamiliar and familiar food [70]. Several previous studies stated that food neophobia concerns mostly choosing or eating new foods and fears of non-traditional foods. Therefore, food neophobia is defined as one of the food-related personality traits and refers to people who are reluctant to try new foods in the food choice process [71]. Food neophobia negatively correlates with not only novel food choices but also foreign food familiarity and unusual experiences related with food. On the other hand, people exhibiting food neophilia are better able to try to taste unfamiliar food items and tend to have a more positive perception of the unfamiliar. Furthermore, unlike food neophobia, those exhibiting food neophilia have less difficulty in engaging in choosing and trying new foods with pleasure [72]. Pliner and Hobden (1992) [71] first tried to evaluate the characteristics of food neophobia, considered food neophobia as one of the personality traits, and suggested a food neophobia scale (FNS) that consists of 10 items. Through their research, many studies have used FNS to assess people’s attitudes and perceptions of new foods, proving that FNS is an effective measure [70,72,73,74]. In Tuorila et al. (1994), [73]’s study, they measured the degree of food neophobia through unfamiliar foreign foods and familiar traditional foods based on the participants’ sensory and verbal information. The results showed that food neophilics had higher preference for all foods than food neophobia. Arvola, Lähteenmäki and Tuorila (1999) [74] also measured how the trait of food neophobia can influence consumer behavior intentions, which are choosing and purchasing familiar and unfamiliar cheeses using the FNS. As a result, the expectation and taste pleasure of food neophobia about both cheeses are lower than neophilia. Kim, Eves, and Scarles (2009) [75] found that physiological factors like food neophilia and food neophobia are among crucial elements influencing consumption of local food in a tourist destination, pointing out that food neophobia may predict the likelihood of future food choice and intake [76]. Furthermore, the authors pointed out that it is necessary to consider the personality traits of food neophobia, which may predict the likelihood of future food intake, in order to investigate foreign, unfamiliar, and exotic food consumption at the destination [2]. From the results of these studies [70,71,72,73,75,76,77], food-related personality traits of visitors influenced visitors’ satisfaction and loyalty. Specifically, Kim, Suh, and Eves (2010) [77] stated that food neophobia can influence tourists’ satisfaction related to food-experience in a destination, and high levels of food neophobia are more likely to hold positive loyalty of food-experience, such as food festivals. 

Kang and Jeong (2008) [78] demonstrated the moderating effects of food neophobia in relationships between food consumption and health concern. The study found that the level of food neophobia (high and low) has moderating effect on health concern and vegetable consumption. In addition, Eertmans et al. (2005) [79] found the moderating effect of food neophobia on food choice motives and food intake, and Chen (2007) [80] revealed that food neophobia has moderating effect on the relationship between food choice motives and attitude to organic food. Most prior research has focused on the situational determinants of neophobic behavior, although there is some evidence for the existence of group differences. In studies related to humans, there are large individual differences in the extent of neophobia [71]. Even though previous studies verified the moderating role of food neophobia among diverse relationships, there is a lack of research that verifies the relationship between various qualities perceived through street food, destination image, and satisfaction. Although a food-related personality trait such as food neophobia is a crucial factor which can have an effect on food-related experience, food choice, and consumer behaviors such as purchasing and recommendation, it is still in its early stage in the tourism and hospitality dimensions. One aim of this study is to identify whether the tendency of food neophobia has a significant effect on tourists’ image of a destination, life satisfaction, and word of mouth. Therefore, the current study set a hypothesis using food neophobia perception about street foods among foreign tourists, and the following hypothesis was established:

**Hypothesis** **10** **(H10).** *The level of food neophobia plays a moderating role in the relationship among experiential quality, destination image, life satisfaction, and word of mouth.*

**Figure 1:**
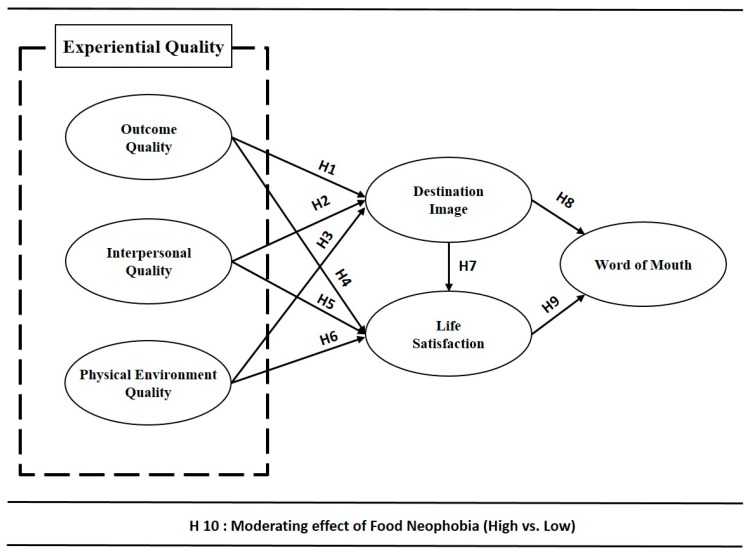
Research model.

The proposed model and the research hypotheses are presented in Figure 1. 

## 3. Materials and Methods

### 3.1. Measurement Items 

A self-administrated questionnaire has been employed for the current study. We conducted a pilot test prior to this study. Based on previous research [33,36,38,39,42,51,61,63,81], a pretest, a pilot test, and developed questionnaire were used to measure the experience quality, destination image, experiential satisfaction, and word of mouth. Then, a pilot test was conducted to assess the adequacy of the measurement through the participation of actual customers in the night market. Slight modification to the items was made based on the results of the pilot test. The first part in the current study included three constructs related to customer experiential quality about street foods in local night markets: outcome, interpersonal, and physical environmental quality. All items from section three to four were measured using a 7-point Likert-type scale (1: strongly disagree to 7: strongly agree) for the following: “Please indicate your level of agreement with the following statement”. To measure the perception of street food experiential quality, this study adopted from Chow et al. (2007) [38] and De Rojas and Camarero (2008) [39], and Kao et al. (2008) [33] studies. Three elements of experiential quality [outcome, interaction, and physical environmental quality] have been estimated using 10 items. Perception of the destination image was measured with four items based on studies by Bigne et al. (2001) [42] and Lee et al. (2005) [51]. Perception of life satisfaction was measured using four items from studies by Oliver (1980) [81], Hellier et al. (2003) [82], Kao et al. (2008) [33] and Wu et al. (2018) [36]. In addition, word of mouth has been modified through Namkung and Jang (2007) [61] and Petrick (2004) [63] studies. Last, this study verified the food neophobia scale using four questionnaires based on Pliner and Hobden (1992) [71] and Ritchey et al. (2003) [72]. The final part of the questionnaire included tourists’ relevant personal information, such as age, gender, ethnicity, and average payment.

### 3.2. Data Collection

An initial survey was modified after pretesting with a sample of three graduate students and faculty members from two universities’ hospitality tourism management department. A pilot test has been conducted to ensure the reliability of the scales using 41 foreign people who had visiting experiences of street food in the night market. The survey was conducted by researchers and a trained interviewer who have distributed and helped to fill questionnaires at nighttime in the three night markets of southern South Korea from May to September 2018. The three street markets (Bupyeong night market and haeundae night market in Busan and kyungju Jungang market in Kyungju), which are located in southern South Korea, were selected by Korea Tourism Association as popular night markets and have various attractive tourism components including street food.

Slight modifications to the questionnaires have been applied to feedback from the pilot test. Each respondent was asked to answer questions based on their experiences of the street foods during their trip. Of the 400 participants, eliminations included 45 questionnaires with missing values and tests for multivariate and univariate outliers found 30 outliers. After all checks for sample validity, 325 responses from participants remained for further hypotheses testing.

### 3.3. Data Analysis

Following the two-step approach reported by Anderson and Gerbing (1988) [83], a confirmatory factor analysis tested whether or not the observed factors reflected the hypothesized latent constructs by using a covariance matrix. The measurement’s reliability was confirmed through calculation of composite reliability. Convergent and discriminant validities have been verified after checking factor loadings and average variance extracted (AVE). After checking the measurement model, structural equation modeling (SEM) is conducted to check the overall fit of the proposed model and evaluate the hypotheses. 

This study employed Structural Equation Modeling (SEM) to compare two models, in both of which the continuous measure of insight was used to predict experiential quality, destination image, life satisfaction, and word of mouth. The formulated model was dichotomized using the same cut-off score as with median dividing the participants into two groups: high food neophobia group and low food neophobia group. This study verified the food neophobia scale using four questionnaires based on Pliner and Hobden (1992) [71] and Ritchey et al. (2003) [72] studies as following. “I do not trust new foods”, “Ethnic foods look too weird to eat”, “I am afraid to eat something I have never had before”, “If I do not know what is in a food, I will not try it” To verify the moderating variable, present study employed median split method. If the indicator value is above the median, the grouping value is “high group”, and if the indicator value is below the median, the grouping value is “low group” [84]. An unconstrained model and constrained model were then analyzed. The effects of insight on experiential quality, destination image, life satisfaction, and word of mouth were constrained to be equal across both groups. In the unconstrained model, these effects were free to differ across the groups. To prove whether or not level of food neophobia exists regarding experiential quality of street food, chi-square differences compared the two models (constrained model versus unconstrained model) with one degree of freedom for each of the three paths’ coefficients. The χ^2^ value of the unconstrained model, which allowed estimating the coefficients in each group without control, was subtracted from the χ^2^ value of the constrained model [85]. 

## 4. Results

### 4.1. Descriptive Statistics

Table 1 shows the results of general characteristics of the subjects. Researcher(s) and assistant(s) visited three major night markets which are the most popular destination to enjoy the street foods for tourists located in southern South Korea. Street markets located in Busan were 64.3% (Bupyeong night market was 39.7% and Haeundae night market was 24.3%), and Kyungju was 36%. Among the 325 valid respondents, 52.3% were male and 47.7% were female. The majority of respondents were Asian (50.8%), followed by Caucasian (26.5%), and African American (11.4%). The companion of more than half of the respondents were either friends (37.2%) or couples (33.2%), and 59.1% of tourists reported that their average payment per visit in the market for street foods were between 5001 to 10,000 won. 

### 4.2. Confirmatory Factor Analysis

This study satisfies convergent validity because all items have relatively high standardized factor loadings on their underlying constructs (values ranged from 0.723 to 0.930), and all are significant at an alpha level of 0.01 (Table 2). In addition, the Cronbach alpha is also greater than standard (0.791 to 0.912) (*p* < 0.001), and all indicators loaded properly on the proposed constructs. To refine all measures for the structural model, assessment of the measurement model used the maximum likelihood estimation method. The results show a proper fit to the data (χ^2^ = 419.853; d.f. = 171; *p* < 0.001; χ^2^/d.f. = 2.455; Root Mean Square Error of Approximation (RMSEA) = 0.067; Goodness-of-Fit Index (GFI) = 0.890; Normed Fit Index (NFI) = 0.927; Incremental Fit Index (IFI) = 0.955; The Tucker–Lewis Index (TLI) = 0.944, and Comparative Fit Index (CFI) = 0.955. Composite reliability was the way for evaluating the instrument’s reliability, as shown in Table 3. Average variance extracted (AVE) is greater than the threshold (0.50) for all constructs [86]. Comparison of AVE for each construct and squared correlations between the paired constructs tested for discriminant validity [87]. Table 3 indicates that AVE for each construct is greater than the squared correlations between paired constructs, demonstrating the discriminant validity.

### 4.3. Structural Model

**Figure 2:**
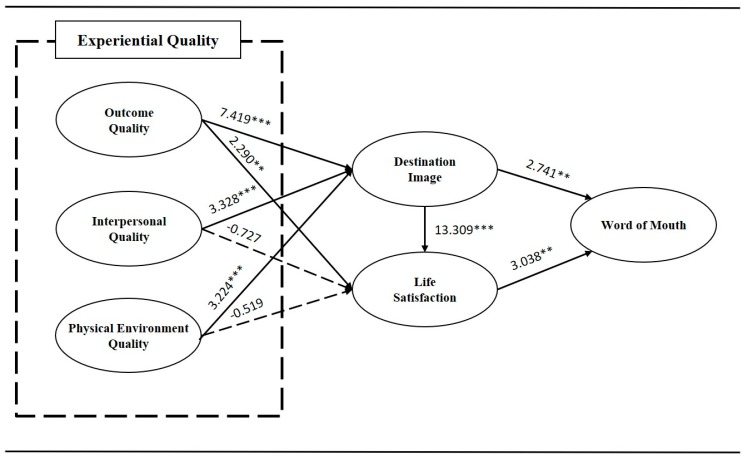
Results of structural equation modeling.

The integrated model shows an adequate fit to the data in the street foods store setting (χ^2^ = 517.376; d.f. = 194; *p* < 0.001; χ^2^/d.f. = 2.677; Root Mean Square Error of Approximation (RMSEA) = 0.072; Goodness-of-Fit Index (GFI) = 0.871; Normed Fit Index (NFI) = 0.915; Incremental Fit Index (IFI) = 0.945; The Tucker–Lewis Index (TLI) = 0.935, and Comparative Fit Index (CFI) = 0.945. Table 4 summarizes the path coefficients for all hypothesized paths in the model, and Figure 2 visualizes the hypothesized paths. All but two of the paths in the formulated model were positive and significant. Therefore, empirical support accrues to all the hypotheses except for Hypotheses 5 and 6, which referred to the path from interpersonal quality to satisfaction and from physical environmental quality to satisfaction. However, except for those two paths, all other paths among experiential quality and destination image and satisfaction were significant. In addition, the current study demonstrates that the relationships among destination image, satisfaction, and word of mouth are statistically significant as well.

Specifically, the results confirm the proposed effects of all experiential qualities (Hypothesis 1: β = 0.553; t = 7.419 ***; Hypothesis 2: β = 0.189; t = 3.238 ***; Hypothesis 3: β = 0.173; t = 3.224 ***). In addition, the present study partially demonstrates the relationship among experiential quality and satisfaction (Hypothesis 4: β = 0.089; t = 2.290 **; Hypothesis 5: β = −0.026; t = −0.727; Hypothesis 6: β =–0.032; t = −0.519). Among the three predictors of experiential quality, outcome quality has the strongest effect on destination image as well as satisfaction. In accordance with the hypotheses, destination image has a direct effect on satisfaction (Hypothesis 7: β = 0.939; t = 13.309 ***), and word of mouth (Hypothesis 8: β = 0.454; t = 2.741 **). Last, the current study has verified the relationship between satisfaction and word of mouth (Hypothesis 9: β = 0.501; t = 3.038 **). Overall, this study produces critical support for the notion that experiential quality (outcome, interpersonal, and physical environment quality), as perceived by tourists during their street food consumption experience, positively influenced their perceptions of destination image and that this, in turn, has a positive effect on travelers’ positive intention to vouch (word of mouth) for street foods in the night market.

### 4.4. Moderating Effects

**Figure 3:**
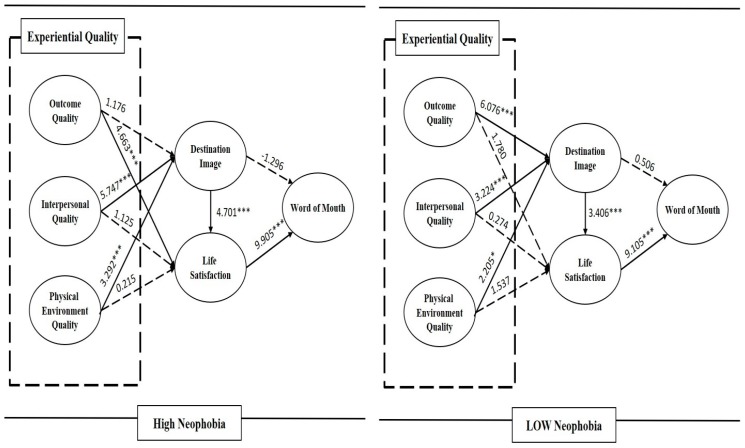
Moderating effect of food neophobia.

This study averaged the answers to four food neophobia scale questions and found the median score (Mean = 4.43; SD = 0.61; Median = 4.50). Therefore, this study identified two different groups, high neophobia group (above 4.50) and low food neophobia group (below 4.49). In order to examine whether or not perception about food neophobia moderates the relationship among experiential quality, destination image, satisfaction, and word of mouth, comparison of two models (constrained model vs. unconstrained model) tested all path coefficients. The difference of chi-square values between the constrained model and unconstrained model was calculated by considering differences of one degree of freedom (Table 5). Significant differences in the chi-square statistic appear for two of the nine individual paths: outcome quality → destination image (Δχ^2^ = 8.417; Δd.f. = 1; *p* < 0.001) and outcome quality → life satisfaction (Δχ^2^ = 5.838; Δd.f. = 1; *p* < 0.01). Especially, outcome quality has the highest difference between the two groups based on the chi-square difference. Results show that the quality outcome has significantly influence on destination image only among the low-food-neophobia group. This study measured the quality outcome through general quality of street food such as taste, proper amount, and attractive appearance. In other words, some tourists who have low street food neophobia formed significant and positive destination images. High food neophobia group, on the other hand, did not form a significant destination image through food-related outcome quality. Namely, people with high objection to street foods found it difficult to form positive destination images through food. In addition, this study found that the outcome quality has significant influence on life satisfaction only for high food neophobia group. In other words, the group with a high level of new objection or fear of food showed higher satisfaction with life through the quality of food than the lower group. Therefore, the present study revealed that the multi-group analysis found structural differences in the model, in particular in the way travelers perceive different perceptions about outcome quality among the destination image and life satisfaction about street foods in a night market according to food neophobia. Therefore, Hypothesis 10 gains partial support. Figure 3 shows the results of moderating effect of food neophobia.

## 5. Conclusions

According to the results of structural equation modeling, there are statistical significances in the relationships among experiential quality, destination image, life satisfaction, and word-of-mouth (WOM). In hypotheses 1–6, these are about the relationship among experiential quality (outcome quality, interpersonal quality, and physical environment quality], destination image, and life satisfaction. The meaning of these results is that outcome quality among experiential quality has the strongest effect on destination image as well as life satisfaction. Some previous studies have also suggested that outcome quality/food quality has a significant impact on restaurant/place/destination image and consumer satisfaction [31,33,37,38,39,43]. In hypotheses 7 and 8, the destination image is shown to be a significant factor in the satisfaction and word of mouth effect. Previous studies explained that the results were similar to the hypothesis test [42,51,53,56,57,58,59]. In hypothesis 9, similar to the results of previous studies [59,60,61,62,63,64,65,66,67,68], satisfaction, also an important predictor of word of mouth, is one of the positive tourists’ behaviors. In summary, this study states that three experiential qualities that positively evaluated Korea’s night market street food had a positive effect on destination image as well as satisfaction. Moreover, the positively assessed destination image and satisfaction were found to have a good influence on word of mouth.

According to the results of moderating effect of food neophobia, there are statistically significant differences between the high neophobia model and low neophobia model in hypotheses 1 and 4. More specifically, in hypotheses 1 and 4, where outcome quality included in experiential quality was an independent variable, level of food neophobia was found to play a role of moderating effect. In hypothesis 1, tourists who are likely to have low levels of food neophobia perceived that outcome quality of street food is a crucial factor in having positive destination image, but tourists with a high level of food neophobia did not. In contrast, looking at hypotheses 2 and 3 together, tourists with high neophobia characteristics perceive that interpersonal quality and physical environmental quality have a significant effect on the destination image. This indicates that food neophobia has a reluctance and fear of new foods, such as foreign food and non-traditional food, so it is not possible to evaluate the image of the countries they visited simply by food quality. Therefore, when they are experiencing street food in Korea, they already have preconceptions, so the environment and service factors are considered more important than outcome quality related to the food in evaluating the image of the Korea they visited. This is similar to the results of previous studies that food perceptions and characteristics of people with food neophobia tend to play an important role in food selection [77,78,79,80].

In hypothesis 4, we measured the influence of the outcome quality on the life satisfaction. This result showed that tourists who have a tendency for a high level of food neophobia perceived that outcome quality of street food has a positive effect on life satisfaction, but tourists with a low level of food neophobia did not. However, looking at hypotheses 5 and 6 together, tourists with a high level of neophobia characteristics assessed that only quality outcome is an important factor affecting satisfaction, not the interpersonal quality and physical environmental quality. This result is the same as the effect of the whole model before comparing the two models to see the moderating effect of food neophobia. To explain this result in detail, due to the locational characteristics of street foods, most of which are located in the middle of markets or streets, environmental factors such as facilities, ambience and lighting are not adequately equipped like restaurants. Therefore, physical environmental quality did not significantly affect satisfaction. Interaction quality also was not an important predictor of satisfaction. Unlike general restaurants and markets, most of the places that provide street food are made up of only one or two staff members and have few seats available. Therefore, there are very few contact points between street food providers and users, and very little time for communication. For example, calculations and orders are done at the same time, and most bring their own food. Because of that, it is rare to deliver food, give a detailed description of the menu, or complain about it. Consequently, because of these characteristics of street food, they focus more on food than interpersonal quality and physical environmental quality, and consider it an important evaluation factor. Therefore, similar to the results of previous studies, the better the street food experience, the higher the overall satisfaction [37,38,39,43].

The hypotheses 7–9 show the relationship between destination image and life satisfaction and word of mouth. It was partially significant in the overall model. Although there was no significant difference between the two models according to the level of food neophobia, the overall model showed a significant influential relationship. Our research results indicated that if the image of Korea recognized by tourists who experienced Korean street food is good, satisfaction is increased. We also found that tourists who had high satisfaction produced word of mouth effects such as recommending to others. This result is similar to the results of previous studies [56,57,58,59,60,61,62,63,64,65,66,67,68]. In summary, our study proved that experiential quality has a significant effect on destination image and satisfaction, as well as a significant relationship between destination image, life satisfaction, and word of mouth. In addition, it was also found that there were differences according to the level of food neophobia, and the results were similar to those of previous studies.

## 6. Discussion and Implications

The present study aimed to identify the influence on tourist experience quality of street foods on destination image, satisfaction, and word-of-mouth in Korea and the moderating impact of food neophobia on the relationship. More specifically, we set and proved 10 different hypotheses: (1–3) influence of experiential quality (outcome quality, interaction quality, physical environmental quality) on destination image, (4–6) influence of experiential quality (outcome quality, interaction quality, physical environmental quality) on life satisfaction, (7–8) influence of destination image on satisfaction and word of mouth, (9) relationship between satisfaction and word-of-mouth, (10) the moderating role of the level of food neophobia in the relationship among experiential quality, destination image, satisfaction, and word-of-mouth. In the tourism and hospitality field, there is little research that investigated not only the relationship among experience quality of street foods on destination image, satisfaction, and word of mouth but also the moderating role of food neophobia. We expanded several concepts from origins to our research model. First, we expanded from service quality to experiential quality of street food related experience in order to assess tourists’ perception and satisfaction. We used different elements in order to measure experience quality, for which we also brought and revised some variables to measure service quality [31,33,37,38,39]. Second, we also brought from consumer satisfaction to satisfaction of tourists who have experienced Korean street food. Moreover, we found that experiential quality has been a predictor that positively influenced experiential value and satisfaction as did some of previous studies in the service sector [31,33,37]. Last, we measured the differences in the tourists’ experiential quality of street food, Korean image, perceived satisfaction, and willingness to recommend to others according to the level of food neophobia. The results of our study were similar to these studies’ results [70,71,72,73,77,78,79,80] in that food-related personality traits of visitors had an influence on visitors’ satisfaction and loyalty. Therefore, based on the results of this study, we proposed managerial implications for food enterprises and food tourism organizers related to street food.

This study contributes to the understanding of how travelers’ experience of Korean street food interacting with experiential quality, destination image, life satisfaction, and word of mouth. Furthermore, we looked at how the relationships differ depending on the tourists’ level of food neophobia. The results of this study can provide insightful guidance for street food marketers and related food product companies. Street food marketers may consider the various characteristics and responses of people (tourists/consumers) with not only general consumers but also some people who have a tendency to food neophobia when they design strategies. First, based on the results of this study, when you think of everybody as a potential consumer, companies and workers related to street food should focus more on the street food itself than on the physical environment and interpersonal factors. For example, if using fresher ingredients for street foods and reducing the use of artificial seasonings to improve nutritional quality, people will be more interested in and satisfied with street foods, which are not inferior to the quality of foods in professional restaurants. In addition, if information about the characteristics of food, flavor, and nutrition of street food are provided and hygiene is maintained, people will perceive the street food as a more reliable and safe food. Second, in order to target the food neophobia consumption market, it is necessary to understand food habits or the social and cultural background of food neophobia. However, novelty about street food still affects tourist satisfaction. Therefore, street food providers in the destination together with other tourism sectors should pay attention to giving information about street foods of Korea. For example, providing not only basic information of street food ingredients, such as types and origins, but also nutritional information such as calories, macronutrients, and fat helps food neophobes reduce their fear of new foods and attempt to eat it. This information can be explained directly by the street food provider when the food is served, or it can be written down on the menu. It is also important to give an impression of some familiarity to street foods in Korea. For instance, marketers may focus on how their street foods can be made to appear more familiar and adaptable to street food markets by using well-known spices and familiar food ingredients. Higher experiential quality perceived by experience could lead to both higher satisfaction levels and more affirmative behavioral intentions in common [42,51]. Therefore, it is also helpful to have customers try to eat novel food more easily by creating an opportunity to encounter new food through events that can be perceived as being favorable to people, such as the provision of a high number of free coupons and free samples [84]. In addition, this study confirms the promising impacts of street food in the destination as predicting tourists’ satisfaction and word of mouth. In sum, travelers perceive their experiences about street food in the destination as valuable when their satisfaction is high and, in this way, their revisit intention or willingness to recommend is increased.

Nevertheless, there are some limitations with this study. First, the sample of participants is narrow. For instance, more than half the participants were composed of Asians. According to previous studies, there is a difference in FNS (Food Neophobia Scale) for each country such as America, Canada, Australia, and so on [71,73,88]. Therefore, it is necessary to adjust the ratio of country composition. Second, the differences of demographic factors in the relationships among experiential quality, destination image, satisfaction, and word of mouth were not examined. Among various demographic factors, age, nationality, and income factors have been mentioned in previous studies. A previous study showed that the younger the person is, the easier it is for him or her to try new foods. In a study that looked at the differences in income, the smaller the income, the more people tended towards neophobia. This is because the lower the income, the less chance they have to encounter new foods (foreign foods, non-traditional food, etc.), and most of them are reluctant to try novel foods. Moreover, previous studies have shown that food neophobia’s degree varies depending on the nationality of the participants [71,73,88]. Specifically, the participants’ levels of food neophobia were scored in FNS (Food Neophobia Scale). The study found that the average FNS varies from country to country, and detailed and differentiated studies are needed. Therefore, in further studies, it is needed to identify the characteristics of people who show differences according to the level of FNS and to find customized strategies. Last, this study employed the structural equation modeling method, and the multitude of parameters (e.g., factor loadings, variances, path coefficients) corresponding to diverse hypotheses are tested simultaneously, and the empirical relationships among observed variables can be repeated by the model in structural equation model. This is only possible if the empirical data deliver enough information to assess all parameters. Namely, structural equation modeling is not based on raw data. Therefore, it is hard to estimate more model parameters than distinct entries in the empirical covariance matrix [89,90].

## Figures and Tables

**Table 1:** General characteristics of the subjects.

Characteristics		n	%
Street Market[s]	Bupyeong Night Market (Busan)	129	39.7
	Haeundae Night Market (Busan)	79	24.3
	Kyungju Jungang Market (Kyungju)	117	36.0
Gender	Male	170	52.3
	Female	155	47.7
Ethnic	African American	37	11.4
	Hispanic	8	2.5
	Asian	165	50.8
	Caucasian[White]	86	26.5
	Other	29	8.9
Companion	Family	53	16.3
	Couple	108	33.2
	Friend	121	37.2
	Colleague	18	5.5
	Alone	22	6.8
	Others	3	0.9
Average payment per visit	Under 3000	10	3.1
	3001–5000	91	28.0
	5001–10,000	194	59.7
	10,001–20,000	30	9.2
Total		325	100

**Table 2:** Constructs and Scale of Items.

Constructs and Scale Items	Mean	SD	Cronbach α ^a^	Factor Loading ^b^
**Experiential Quality**				
*Outcome Quality*			**0.791**	
I had an excellent experience of street food while staying in the food market.	5.10	0.987		0.793
I am impressed by the street food quality in the food market.	5.28	1.053		0.735
I felt positive things about the street food products for travelers.	5.18	1.035		0.883
*Interpersonal Quality*			**0.902**	
The employees were kind and supporting in street food market.	4.83	1.224		0.746
The interaction I had with the staff was of a high standard.	4.70	1.248		0.892
I feel good about the interaction I had with the employees in street food market.	4.77	1.235		0.878
Overall, I would say the quality of my interaction with the employees was excellent in the street food market.	4.82	1.216		0.857
*Physical Environmental Quality*			**0.839**	
The physical environment in this street food store is excellent.	4.77	1.280		0.728
I am impressed with the general quality of this street food markets’ physical atmosphere.	5.11	1.232		0.757
I believe that the environment of street food stores in the night food market was of high quality.	4.59	1.359		0.915
**Destination Image**			**0.883**	
The place for the street food provides good service.	4.86	1.128		0.802
The place for the street food gave a positive image of the destination.	4.50	1.330		0.825
The place for the street food has good reputation.	4.07	1.376		0.824
The place for the street food helped to get a good image of my trip.	4.10	1.588		0.808
**Life Satisfaction**			**0.932**	
The street food in the night market is beyond my expectations.	4.65	1.212		0.917
I really like this trip to this street food market.	4.84	1.185		0.813
The experience of street foods makes me happy about my trip.	4.75	1.148		0.886
It is worthwhile to stay in here.	4.88	1.118		0.883
**Word of Mouth**			**0.912**	
I will say positive things about this street food to other people.	5.10	1.296		0.930
I would highly recommend the street food in the night market to my friends and relatives.	4.58	1.378		0.881
If I could, I would recommend the street food.	4.87	1.237		0.839

Note 1. SD = Standard Deviation. Note 2. ^a^ Bold figures display Cronbach Alpha. Note 3. ^b^ All factor loadings are significant (*p* < 0.001).

**Table 3:** Confirmatory Factor Analysis and Discriminant Validity.

	OUT	INT	PHY	DESI	LSA	WOM
OUT	0.556 ^1^					
INT	0.264	0.714				
PHY	0.141	0.115	0.647			
DESI	0.496	0.283	0.202	0.664		
LSA	0.450	0.318	0.171	0.812	0.767	
WOM	0.500	0.277	0.158	0.903	0.850	0.782
χ^2^ = 419.853, d.f. = 171, GFI = 0.89, AGFI = 0.85, NFI = 0.92, RFI = 0.91, IFI = 0.96, TLI = 0.94, CFI = 0.96, RMSEA = 0.067						

Note 1. OUT = Outcome clue; INT = Interaction clue; PHY = Physical Environmental Clue; DESI = Destination Image; LSA = Life Satisfaction; WOM = Word of Mouth; IFI = Incremental Fit Index; TLI = Tucker–Lewis Index; CFI = Comparative Fit Index; GFI = Goodness-of-Fit Index; RMSEA = Root Mean Square Error of Approximation. Note 2. ^1^ AVE is on the diagonal. Squared of paired constructs are on the off-diagonal.

**Table 4:** Structural Parameter Estimates.

Hypothesized Path	Coefficient	t-Value	Results
Hypothesis 1: OUT→DESI	0.553	7.419 ***	Supported
Hypothesis 2: INT→DESI	0.189	3.238 ***	Supported
Hypothesis 3: PHY→DESI	0.173	3.224 ***	Supported
Hypothesis 4: OUT→ESA	0.089	2.290 **	Supported
Hypothesis 5: INT→LSA	−0.026	−0.727	Not Supported
Hypothesis 6: PHY→LSA	−0.032	−0.519	Not Supported
Hypothesis 7: DESI→LSA	0.939	13.309 ***	Supported
Hypothesis 8: DESI→WOM	0.454	2.741 **	Supported
Hypothesis 9: LSA→WOM	0.501	3.038 **	Supported
χ^2^ = 517.376, d.f. = 194, GFI = 0.87, AGFI = 0.83, NFI = 0.92, RFI = 0.90, IFI = 0.95, TLI = 0.94, CFI = 0.95, RMSEA = 0.072			

Note 1. * *p* < 0.05, ** *p* < 0.01, *** *p* < 0.001. Note 2. OUT = Outcome clue; INT = Interaction clue; PHY = Physical Environmental Clue; DESI = Destination Image; LSA = Life Satisfaction; WOM = Word of Mouth.

**Table 5:** Moderating effects of level of food neophobia (low and high].

Hypothesized Path	Unconstrained χ^2^	Constrained χ^2^	t-Value		Model Comparison Δχ^2^ (Δd.f. = 1)
			Low	High	
Hypothesis 1: OUT→DESI	658.554	667.024	6.076 ***	1.716	8.471 ***
Hypothesis 2: INT→DESI	658.554	660.607	3.224 ***	5.747 ***	2.053
Hypothesis 3: PHY→DESI	658.554	659.008	2.205 *	3.292 ***	0.454
Hypothesis 4: OUT→LSA	658.554	664.392	1.780	4.663 ***	5.838 **
Hypothesis 5: INT→LSA	658.554	659.710	0.274	1.125	1.156
Hypothesis 6: PHY→LSA	658.554	659.347	1.537	0.215	0.793
Hypothesis 7: DESI→LSA	658.554	659.608	3.406 ***	4.701 ***	0.055
Hypothesis 8: DESI→WOM	658.554	660.238	0.506	−1.296	1.684
Hypothesis 9: LSA→WOM	658.554	659.304	9.105 ***	9.905 ***	0.750

Note 1. * *p* < 0.05, ** *p* < 0.01, *** *p* < 0.001.
